# Glutathionylation on RNA-binding proteins: a regulator of liquid‒liquid phase separation in the pathogenesis of amyotrophic lateral sclerosis

**DOI:** 10.1038/s12276-023-00978-2

**Published:** 2023-04-03

**Authors:** Hyun-Jun Choi, Ji Young Lee, Kiyoung Kim

**Affiliations:** 1 Soonchunhyang Institute of Medi-bio Science, Soonchunhyang University, Cheonan, 31151 Korea; 2 Department of Integrated Biomedical Sciences, Soonchunhyang University, Cheonan, 31151 Korea; 3 Department of Medical Biotechnology, Soonchunhyang University, Asan, 31538 Korea; 4 Department of Medical Science, Soonchunhyang University, Asan, 31538 Korea

**Keywords:** Protein aggregation, Neurodegeneration

## Abstract

RNA-binding proteins (RBPs) containing low-sequence complexity domains mediate the formation of cellular condensates and membrane-less organelles with biological functions via liquid‒liquid phase separation (LLPS). However, the abnormal phase transition of these proteins induces the formation of insoluble aggregates. Aggregates are pathological hallmarks of neurodegenerative diseases, such as amyotrophic lateral sclerosis (ALS). The molecular mechanisms underlying aggregate formation by ALS-associated RPBs remain largely unknown. This review highlights emerging studies on various posttranslational modifications (PTMs) related to protein aggregation. We begin with the introduction of several ALS-associated RBPs that form aggregates induced by phase separation. In addition, we highlight our recent discovery of a new PTM involved in the phase transition during the pathogenesis of fused-in-sarcoma (FUS)-associated ALS. We suggest a molecular mechanism through which LLPS mediates glutathionylation in FUS-linked ALS. This review aims to provide a detailed overview of the key molecular mechanisms of LLPS-mediated aggregate formation by PTMs, which will help further the understanding of the pathogenesis and development of ALS therapeutics.

## Brain disease: Understanding protein aggregation in amyotrophic lateral sclerosis

The addition of peptide tags to proteins implicated in neurodegeneration can alter the ways in which disease-causing aggregates form inside brain cells. Kiyoung Kim and colleagues from Soonchunhyang University, Asan, South Korea, review how molecular changes made to RNA-binding proteins associated with amyotrophic lateral sclerosis (ALS) can affect the dynamics by which these proteins assemble inside liquid-like compartments within the cell. One such change, known as glutathionylation, involves the addition of antioxidant peptide tags to proteins such as the RNA-binding protein FUS, a key driver of ALS onset and progression. This process alters the electrical charge of FUS, changing the way it interacts with other proteins, and reducing its propensity to form disease-promoting aggregates. A better understanding of this process could aid in the treatment and management of ALS and related brain diseases.

## Introduction

Amyotrophic lateral sclerosis (ALS) is a fatal neurodegenerative disease characterized by the progressive and selective degeneration of upper and lower motor neurons in the spinal cord^1–3^. Approximately 90% of ALS cases are sporadic ALS, with less than 10% being inherited (familial ALS). Familial ALS is strongly associated with family history and genetic causes of the disease^4^. ALS pathogenic mutations identified in RNA-binding proteins (RBPs) include TAR DNA binding protein 43 (TDP-43), fused-in-sarcoma (FUS), Ewing sarcoma (EWS), TATA-binding protein-associated factor 15 (TAF15), and heterogeneous nuclear ribonucleoproteins (hnRNPs)^5,6^. These RBPs form aggregates in the cytoplasm of motor neurons and sometimes in other cell types, such as glial cells, eventually leading to neuronal cell death and toxicity^7–10^.

**Fig. 1:**
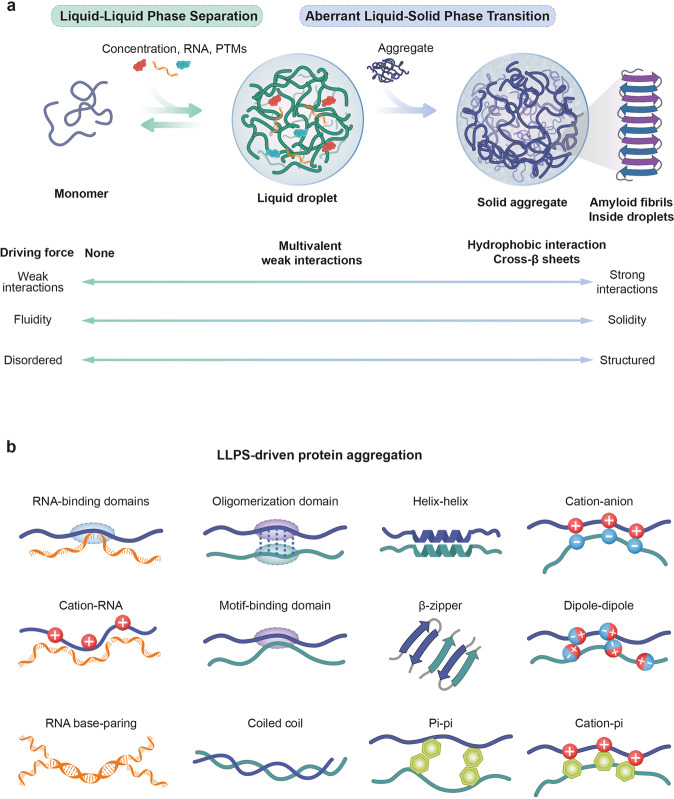
Protein phase transition and driving force. **a** The liquid-like protein condensates formed through liquid‒liquid phase separation (LLPS) are highly dynamic and constantly exchange with the surrounding environment. With time and changes in the surrounding environment, the solidification of liquid-like condensates to hydrogels and amyloid fibrils occurs via liquid‒solid phase transition (LSPT). **b** Various types of multivalent interactions that promote the initiation and maintenance of LLPS include RNA-binding domains, oligomerization domains, motif-binding domains, helix-helix interactions, β-zippers, π–π interactions, cation‒anion interactions, dipole‒dipole interactions, and cation–π interactions.

For decades, researchers have investigated the unifying mechanisms responsible for the complex pathogenesis of ALS. Liquid‒liquid phase separation (LLPS) has recently been implicated in major pathways involved in the pathogenesis of ALS. LLPS is a reversible phenomenon that decomposes a homogenous solution into two contemporaneous liquid phases with a dense phase (resembling liquid droplets) and a dilute phase through intermolecular interactions^11–15^. Protein aggregation may originate from LLPS (Fig. 1a).

LLPS generates cellular condensates and membrane-less organelles (MLOs) with biological functions. LLPS also feature less well-characterized compartments from the external environment due to a deficiency of lipid membranes. MLOs are composed of macromolecules, such as proteins and nucleic acids^14,15^, and are localized within the cytoplasm and nucleus^11,12,16,17^. Sites include the nucleolus, paraspeckles, nuclear speckles, Cajal bodies, and promyelocytic leukemia bodies in the nucleus and P-bodies, stress granules, germ granules, and mRNA granules in the cytoplasm^12,13,16,18^. Furthermore, the physicochemical properties of LLPS enable MLOs to exhibit liquid-like properties, such as fluidity, dynamics of formation and dissolution, partitioning ability, and diffusional properties. These properties support distinct cellular functions of MLOs, including cell stability, division^19^, proteolysis^20^, gene expression regulation, RNA metabolism, homeostasis^21^, mitochondrial ribosome biogenesis, and mRNA processing^22^. The formation of the condensed phase via LLPS is a dynamic, reversible, and nontoxic process. Further phase transition, called liquid‒solid phase transition (LSPT), results in the formation of solid aggregates from liquids via hydrophobic interactions between molecules. These solidified aggregates are irreversible, and usually toxic. Eventually, aggregated proteins enter the amyloid state by polymerizing into linear structures with ordered cross-sheet connections (Fig. 1a). LLPS has been implicated in cancer and many neurodegenerative diseases, such as Alzheimer’s disease (AD), ALS, and frontotemporal dementia (FTD)^11,23–27^. However, the molecular mechanisms by which LLPS induces gel and aggregate transitions remain largely unknown.

Posttranslational modifications (PTMs) are widely found in eukaryotic cells. These modifications enhance the structural and functional diversity of the proteome via the covalent attachment of functional groups, proteolytic cleavage, or the degradation of entire proteins. More than 200 different types of PTMs have been reported^28^. Other recent studies have suggested that PTMs are correlated with LLPS^29–32^. PTMs can stimulate or counteract phase separation and protein aggregation depending on their charge, modified amino acid residues, and position in the target proteins^32^. In addition, PTMs regulate interactions with other cellular molecules, including proteins, nucleic acids, lipids, and cofactors. Thus, LLPS can likely be modulated by the regulation of PTM (Fig. 1a).

In this review, we discuss how PTMs of RBPs influence the formation of cytoplasmic protein aggregates by phase separation in the pathogenesis of ALS and summarize recent studies describing the effects of PTMs in RBPs during the pathological phase transition of ALS. Furthermore, we focus on the role of protein glutathionylation recently found in fused-in-sarcoma (FUS)-associated ALS and discuss whether this glutathionylation has a pathological role in the development and progression of ALS by modulating LLPS of RBPs, particularly FUS.

## The driving force of phase separation

In physics, phase separation occurs when a molecule reaches its upper limit of dissolution or when the entropy of a solution is maximally maintained, pushing the molecules out of solution^31,33^. Multiple molecules in solution tend to be distributed with energetically advantageous properties. Molecules that induce LLPS spontaneously form droplets and new liquid phases through phase transitions. What leads to these properties?

The protein that drives LLPS is composed of intrinsically disordered regions (IDRs) that do not have a three-dimensional structure. These regions typically contain only a small number of amino acids and repetitive sequence elements. The sequence composition of IDRs can vary, but it is typically disproportionately represented by only a few amino acids and is referred to as a low-complexity domain (LCD)^34–37^. In addition, prion-like domains (PrLDs) enable specific proteins to form self-propagating amyloid fibers and are rich in hydrophilic amino acids that include asparagine, glutamine, serine, and tyrosine^38–40^. Furthermore, several IDRs of LLPS-associated proteins contain arginine/glycine-rich (RGG-rich) regions in which charged amino acids, such as arginine/glycine, are disproportionately represented^27^. This results in IDR-containing proteins that exhibit a simplified primary structure and have highly flexible and dynamic properties^41^. Thus, they are more exposed to the external environment than other proteins and have more opportunities to interact with other intracellular molecules. The multivalent synergistic effect of weak interactions between amino acid groups and other macromolecules, such as proteins and nucleic acids, contributes to the formation of phase separation^13,42–48^. Among these multivalent interactions, electrostatic interactions are the best known and are especially critical in heterotypic LLPS of protein/RNA mixtures. In addition, π-π stacking of aromatic residues, cation-π interactions between arginine or lysine residues and aromatic side chains (e.g., phenylalanine, tyrosine, tryptophan), dipole‒dipole interactions, hydrophobic interactions, and hydrogen bonding interactions are imporant^17,24,49,50^. Furthermore, diverse adhesive domains/motifs, such as oligomerization domains, coiled coils, and β-zippers, provide multiple intramolecular and intermolecular interactions (Fig. 1b).

## LLPS of ALS-associated RBPs

A number of previous studies have suggested that phase separation is involved in the pathological process associated with ALS. Many proteins are associated with ALS pathogenesis, and mutations in the genes encoding them usually deepen and accelerate LLPS and eventually form fibril aggregates^51–57^. Well-known proteins include FUS, TAF15, TDP-43, and hnRNPs^58,59^. These RBPs play pivotal roles in cells, regulating transcription and translation by interacting with RNA.

### FUS, EWS, and TAF15

FUS, EWS, and TAF15 belong to the FET (FUS/EWS/TAF15) family with similar functions and structures^60^. The domain structure includes the N-terminal region that comprises LCD, a C-terminal domain with an RNA-recognition motif (RRM), several RGG-rich regions, a zinc-finger (ZnF) domain, and a nuclear localization signal (NLS) domain^61,62^. FUS-LCDs are important in mediating both LLPS and the highly reversible formation of fibril aggregates^63^. Moreover, a nuclear magnetic resonance (NMR) study showed that N-terminal FUS-LCD (FUS-LCD-N) forms a fibril core via hydrophilic interactions^64,65^. In addition, the C-terminus of the FUS-LCD domain (FUS-LCD-C) forms fibril aggregates^66,67^. ALS disease-related mutations were reported to significantly accelerate the LLPS of both full-length FUS and FUS-LCD to form cross-β-aggregates, a highly stable fibril^39,64,67,68^. The above process includes weak interactions that act multivalently, such as hydrogen bonding, *π*/*sp*^2^, and hydrophobic interactions^37^. The structures of TAF15 and EWS are similar to the structure of FUS, including PrLD. Therefore, they also form condensates via LLPS^43,69–71^.

### TDP-43

TDP-43 has a multiple-domain structure consisting of an NTD that mediates weak self-interactions^72,73^, two RRMs, an intrinsically disordered CTD that mediates heterotypic interactions with binding partners using glycine-rich LCD, and helix-helix contacts for self-assembly by LLPS^74,75^. In the droplet state, TDP-43 LCD forms monomeric states with the potential for self-aggregation. In contrast, increasing the concentration of TDP-43 LCD leads to more gel-like formations^52^. In addition, protein expression with ALS-associated mutations reduces LLPS and enhances aggregation^52^. These results suggest that ALS mutation interferes with the LLPS of TDP-43 and induces the formation of aggregates.

### hnRNP A1/A2

hnRNPs A1 and A2 are prototypical hnRNPs. They are RBPs that contribute to multiple functions, including splicing regulation, mRNA stabilization, and transcriptional and translational regulation^76^. In addition, previous studies have shown that mutations in the LCD of hnRNP A1 and A2 cause ALS and multisystem proteinopathy^77^. hnRNP A1 and A2 consist of two RRMs in the NTD domain and an intrinsically disordered RGG-rich C-terminal domain. Moreover, hnRNP A1 and A2 exhibit an intrinsic tendency to assemble into amyloid-like fibrils containing cross-β structures, suggesting the mediation of stress granule (SG) assembly^77,78^. hnRNP A1 and A2 mediate LLPS via weak interactions between aromatic residues in LCD. Furthermore, LCD-mediated LLPS contributes to the assembly of SGs and drives pathological fibrillization^34,77^.

## Effect of PTMS on phase separation

IRD-containing ALS-associated proteins can be highly modified through PTMs because they are easily exposed to the external environment owing to a lack of secondary structure^41^. In addition, numerous studies have shown that PTMs of these IRDs can affect variations in covalent, noncovalent, electrostatic, and hydrophobic interactions. This suggests that PTMs on the IDR of LLPS-associated proteins may affect the phase separation tendency (Table 1). In this section, we discuss how PTMs affect the phase separation properties of ALS-associated proteins.

**Table 1:** Summary of ALS-associated RBP PTMs and effects on LLPS.

PTMs	Regulatory effects on LLPS	Proteins	Key findings	Reference
Phosphorylation	Inhibition	FUS	DNA-PK-mediated FUS-LCD phosphorylation and phosphomimetic substitution prevent phase separation and aggregation in vitro and in cells.	^82^
			DNA-PK-induced FUS phosphorylation of FUS disassembles liquid droplets in vitro.	^83^
			Phosphorylation of FUS-LCD disrupts hydrogel binding and liquid droplet formation in vitro.	^64^
		TDP-43	Phosphomimetic substitution (S48E) of TDP-43 NTD effectively disrupts the LLPS of TDP-43 in vitro and in cells.	^84^
			Casein kinase 1δ-mediated hyperphosphorylation and phosphomimetic substitution of TDP-43 CTD reduce TDP-43 phase separation and aggregation in vitro and in cells.	^90^
		hnRNP A2	Fyn-mediated phosphorylation of hnRNP A2 LCD reduces neurodegeneration by inhibiting phase separation and aggregation of hnRNP A2 in *C. elegans* model.	^92^
Methylation	Inhibition	FUS	AdOx-induced hypomethylation of FUS promotes phase separation and gelation in vitro and in cells.	^103^
			FUS methylation disrupts the interaction between FUS and TNPO1, reducing phase separation and SG formation in vitro and in cells.	^104^
		hnRNP A2	PMRT1-induced methylation of hnRNP A2 LCD diminishes LLPS.	^106^
Citrullination	Inhibition	FUS	FUS aggregation is increased in PAD4 knockout mice; PAD4 overexpression-induced citrullination inhibits FUS phase separation in cells.	^113^
Acetylation	Promotion	FUS	NatA-induced FUS-LCD acetylation promotes phase separation in vitro.	^119^
Ubiquitination	Inhibition	UBQLN2	UBQLN2 ubiquitination attenuates phase separation behavior of UBQLN2 in vitro and in cells.	^127^
Poly(ADP-ribosyl)lation	Promotion	FUS, EWS, TAF15	PARP inhibition prevents FET protein recruitment through LLPS in the DNA damage site, while PARG inhibition prolongs the presence of FET proteins in vitro and in cells.	^130^
		TDP-43, hnRNP A1	PARylation promotes stress granule formation and phase separation of TDP-43, hnRNP A1 in vitro and in cells.	^131^
		TDP-43	PAR binding promotes liquid‒liquid phase separation of TDP-43 in vitro and is required for TDP-43 accumulation in stress granules in mammalian cells and neurons.	^132^
Glutathionylation	Promotion	FUS	Glutathionylation of FUS RanBP2-type ZnF domain induces FUS aggregation by promoting phase separation; GstO2 inhibits FUS phase separation and aggregation by reducing glutathionylation in vitro and in *Drosophila* neurons.	^148^

### Phosphorylation

Phosphorylation is the most well-known PTM and is a typical mechanism that controls protein function and transmits cellular signaling throughout the cell. Phosphorylation adds a negatively charged phosphoryl group to the amino acid hydroxide group via a covalent bond. This can change the charge distribution and electrostatic interactions of the target protein. Serine, threonine, and tyrosine residues can be phosphorylated. However, serine and threonine residues are highly distributed in PrLD. Thus, the formation of LLPS and aggregates can be regulated by phosphorylation^79–81^.

Most previous ALS studies have concluded that phosphorylation prevents phase separation in LLPS-associated proteins. For example, Monahan et al. demonstrated that DNA-dependent protein kinase (DNA-PK) phosphorylates 12 sites in FUS NTD-PrLD (T7, T11, T19, S26, S30, S42, S61, T68, S84, S87, S117, and S131), both in vitro and in human cells^82^. Furthermore, phosphomimetic substitution (S/T → E) of the 12 DNA-PK consensus sites inhibits FUS-PrLD-induced phase separation and forms fibrillated aggregates in vitro^82^. Significantly, FUS phosphomimetic substitution reduces aggregation in human cells and yeast models and controls FUS-associated cytotoxicity^82^. Therefore, prior studies have generally suggested that increased FUS-PrLD phosphorylation diminishes aggregation and toxicity^64,82,83^.

TDP-43 is an RBP that is mainly aggregated in patients with ALS and AD. TDP-43 has a globular NTD that forms a linear polymer through low-affinity head-to-tail intermolecular contacts and contributes to TDP-43 phase separation in vitro and in cells^84^. The TDP-43 NTD contains pS48 S/T phosphorylation, which has been detected in multiple phosphoproteomic analyses of various cell lines^85,86^. Wang et al. showed that phosphomimetic substitution (S48E) of NTD can effectively disrupt the LLPS of TDP-43 in vitro and in cells^84^. In addition, TDP-43 has a PrLD on the C-terminal domain that is multiphosphorylated and aggregated in ALS motor neurons. The phosphorylation of S409/410 on the TDP-43 CTD was identified in patients with TDP-43-induced frontotemporal lobar degeneration (FTLD), as well as highly consistent features in pathologic inclusions^87^. However, phosphomimetic substitution (S → D) of serine 409 and 410 on the TDP-43 CTD was reported to significantly reduce TDP-43 aggregation^88^. Hyperphosphorylation of several TDP-43 CTD serine residues via casein kinase 1δ was previously reported occur at disease-associated sites, likely promoting TDP-43 aggregation^89^. Gruijs et al. established that casein kinase 1δ mediates the hyperphosphorylation of the TDP-43 CTD^90^. In the ALS spinal cord, 12 serine phosphorylation sites (S373, S375, S379, S387, S389, S393, S395, S403, S404, S407, S409, and S410) were identified on TDP-43 CTD by mass spectrometry^91^. Phosphomimetic substitution (S/T → D/E) of these sites reduced TDP-43 phase separation and aggregation in vitro and in cells, endowing the TDP-43 condensate with more dynamic and liquid-like properties^90^. Moreover, the data from multiscale molecular dynamics simulations suggest that suppression of phase separation is associated with the loss of protein‒protein interactions in the TDP-43 CTD and enhanced solvation of negatively charged groups^90^.

hnRNP A2 is similar to other RBPs, including hnRNP A1, FUS, and TDP-43, and is a well-known cause of ALS. In another example, Ryan et al. established that tyrosine can phosphorylate (pY) hnRNP A2, involving approximately four to eight phosphorylated tyrosine residues of LCD. The authors reported that hnRNP A2 LCD tyrosine phosphorylation can alter phase separation and inhibit the formation of hnRNP A2 aggregation in vitro and in vivo, as well as disease ALS/FTD-associated mutants (hnRNP A2-D290V)^92^. Furthermore, the Fyn tyrosine kinase that phosphorylates hnRNP A2 LCD reduces neurodegeneration by inhibiting phase separation and aggregation of hnRNP A2 in a *Caenorhabditis elegans* model^92^. Some studies have demonstrated that phosphorylation induces electrostatic repulsion by adding negatively charged molecules as a common mechanism of LLPS negative regulation by phosphorylation. It notably diminishes the weak intermolecular interactions and improves protein–water interactions, which deter the aggregation of RBPs and promote the dissolution of the preformed fibril aggregation^82,93,94^.

### Methylation

Arginine is an amino acid with a positive charge that mediates hydrogen bonding and amino-aromatic interactions. Arginine methylation is an abundant PTM in which a methyl group is added to the arginine residues of a protein to modify recognition by binding partners or to modulate their biological activity^95,96^. This process is mediated by protein arginine methyltransferases (PRMTs), which catalyze methylation to modify the guanidino nitrogens of the arginine residue by adding one or two methyl groups^95^. Methylation does not change the net positive charge of the arginine residue. However, it is hydrophobic and reduces the hydrogen bond potential, thereby altering interactions between other molecules, including proteins and nucleic acids^97–99^. Importantly, arginine methylation can occur within the RGG/RG motif, in which the arginine-rich domain modifies RNA binding and LLPS^99^. Similar to phosphorylation, arginine methylation is known to inhibit the LLPS of ALS-associated proteins. In FUS, numerous arginine residues are located in the arginine-rich CTD, which are crucial for the phase separation of FUS and are highly methylated in mono- or dimethylated forms^100^. However, in FUS-induced ALS/FTD, FUS is hypomethylated and accumulates in neurons as both nuclear and cytoplasmic aggregates. This occurs in the same manner as EWS and TAF15^101,102^. These observations suggest that arginine methylation may physiologically control FUS-induced phase separation via variance in physiological interactions.

Qamar et al. described the induction of arginine hypomethylation of FUS using adenosine-2,3-dialdehyde, an extensively used inhibitor of arginine methyltransferase activity. The aim was to evaluate the effects of reduced methylation on FUS phase behavior. Importantly, FUS hypomethylation strongly promoted phase separation and gelation in vitro and in cells^103^. The suggested mechanism is intermolecular β-sheet hydrogen bonding and cation-π interactions between C-terminal arginine residues and N-terminal tyrosine residues, which drive the phase separation of FUS. However, arginine methylation increases hydrophobic features while reducing hydrogen bond potential, resulting in weakened cation-π strength and impaired interactions between other molecules, ultimately inhibiting the phase separation of FUS^103^.

In addition, Hofweber et al. showed that arginine methylation-mediated interactions between FUS and the molecular chaperone TNPO1 reduce the LLPS and SG association of FUS. Loss of FUS arginine methylation, as in ALS/FTD patients, elevates LLPS and SG formation of FUS and consequently may contribute to FUS aggregation in ALS/FTD patients^104^.

hnRNP A2-LCD also induces LLPS and is converted to aggregates. hnRNP A2-LCD contains RGG repeats, which are IDRs. Furthermore, some studies have shown that hnRNP A2-LCD is methylated by PRMT1^105^. Ryan et al. also established that hnRNP A2-LCD undergoes LLPS, and disease-associated mutations (P298L and D290V) induce aggregation in vitro. However, the PMRT1-induced methylation of arginine residues located at the RGG site in hnRNP A2-LCD was reported to markedly diminish LLPS^106^. Data from molecular dynamics simulations suggest that the leading cause of this phenomenon is the dimethylation of hnRNP A2 LCD, which inhibits arginine-aromatic interactions, thereby reducing LLPS^106^.

### Citrullination

Citrullination also occurs in arginine residues. Instead of the addition of a functional group, the arginine side chain undergoes oxidation or deimination. In this reaction, peptidyl arginine deiminases (PADs) catalyze the cation-π interaction oxidation of an imine group (=NH), forming a ketone group (=O)^107–109^. In this reaction, the positively charged side chain of arginine is hydrolyzed by water to form neutral urea, leaving a neutrally charged amino acid^110^. This shift in charge can affect protein‒protein interactions and hydrogen bond formation^111,112^.

Interestingly, several consensus sites for PAD have been identified in RG/RGG motifs. These sites are associated with the phase separation of RBPs, including the FET family^113^. The citrullination of FUS via PAD4, a PAD identified in humans, reduces FUS recruitment to SGs^113^. Importantly, PAD4-mediated citrullination significantly inhibits the aggregation of FET proteins^113^. The authors also reported that mouse embryonic fibroblasts of PAD4 knockout mice showed increased FUS aggregation and sequestration into SGs compared with PAD4 overexpression cells, indicating that citrullination inhibits FUS phase separation^113^. Intermolecular β-sheet hydrogen bonding and cation–π interactions between C-terminal arginines and N-terminal tyrosines modulate FUS phase separation^103^. However, when citrullination occurs, the positive charge of the arginine side chain is removed, and intermolecular interactions can be disrupted by altering hydrogen bonds and cation-π interactions, ultimately inhibiting FUS phase separation.

### Acetylation

Acetylation is a major PTM in which an acetyl group is transferred from acetyl-coenzyme A (acetyl-CoA) to the ε-amino side chain of a lysine residue within a protein, regulating phase separation. These reactions are catalyzed by lysine acetyltransferase (KAT), and the reverse process is regulated by lysine deacetylase (KDAC), while the acetyl group can add to the amino acid at the N-terminus via N-terminal acetyltransferases (NATs)^114,115^. This leads to the neutralization of their positive electrostatic charge, which may influence protein interactions with substrates, cofactors, and other macromolecules^116–118^.

The effect of acetylation on phase separation in ALS has not been well studied. However, Bock et al. found that NatA, an N-terminal acetyltransferase, can acetylate FUS-LCD. Importantly, N-terminal acetylation promoted phase separation and reduced the aggregation of FUS-LCD in vitro. Despite N-terminal acetylation, the authors did not observe a significant shift in the structure of FUS-LCD^119^. The authors suggested that the neutralization of FUS-LCD by N-terminal acetylation may disturb other interactions between the molecules as well as phase separation by changing the peptide net charge from −2 to −3^119^.

### Ubiquitination

Ubiquitination is an essential PTM mediated by the ubiquitin (Ub)-conjugating system, which is composed of the E1 Ub-activating enzyme, E2 Ub-conjugating enzyme, and E3 Ub ligase. Ubiquitination leads to covalent attachment of Ub, typically to lysine residues on target proteins, which eventually leads to monoubiquitination or polyubiquitination^120^. Ubiquitination is strongly involved in the Ub-proteasome system (UPS), a crucial protein degradation system in eukaryotes. In these processes, Ub or polyUb attached to target proteins marks them as substrates of proteasomes for degradation^121^. Moreover, abnormal UPS function has been observed in several human diseases, including cancer and neurological diseases^122,123^. However, the correlation between ubiquitination and the phase separation mechanism has not been clearly explained.

Recent studies have suggested that ubiquitinated proteins can regulate phase separation, and a link between Ub and phase separation is emerging^124^. Ub-like protein ubiquilin 2 (UBQLN2) is a proteasomal shuttle factor that is essential for cellular protein quality control. UBQLN2 is expressed in many human tissues, with the highest expression levels in the nervous system^125^. Mutations in *UBQLN2* have recently been shown to cause dominant X-linked inheritance of ALS/dementia^126^. Dau et al. found that UBQLN2 colocalizes with SGs under cellular stress conditions in vivo and undergoes LLPS in vitro^127^. Interestingly, noncovalent Ub or polyUb binding attenuated UBQLN2 phase separation^127^. NMR analysis suggested that LLPS is driven by multivalent interactions of polar or hydrophobic residues on UBQLN2. However, when Ub binds to the UBA domain, it inhibits LLPS by disrupting only UBQLN2 multivalent interactions^127^.

### Poly(ADP-ribosyl)lation

Poly(ADP-ribose) (PAR) is a polyvalent, highly negatively charged, nucleic acid-like polymer. Poly ADP-ribosylation, also known as PARylation, is a type of PTM in which polymers of ADP-ribose are covalently attached to target proteins by PAR polymerase (PARP) enzymes, resulting in a dramatic electrostatic change of the acceptor protein surface^128^. ADP-ribose can be attached to serine, lysine, arginine, aspartate, or glutamate residues by PARPs and is reversible by PAR glycohydrolase (PARG)^128^. In addition, PARylation has been strongly implicated in SG formation, suggesting that it might be a critical modifier for the dynamic assembly/disassembly of disease-related RNP granules, including disease-related RBPs, such as the FET family and TDP-43^129^. IDR-containing proteins, such as FUS, EWS, and TAF15, accumulate through LLPS at sites of DNA damage in a PAR-dependent manner^130^. Moreover, PARP inhibition reportedly prevented the recruitment of FET proteins, while PARG inhibition prolonged the presence of FET proteins in DNA damage sites in vitro, which was also observed in hnRNP A1 and TDP-43^131^.

McGurk et al. found that the downregulation of the tankyrase PARP reduced cytoplasmic TDP-43 accumulation and potently attenuated neurodegeneration in a *Drosophila* model^132^. The authors also described the noncovalent binding of TDP-43 to PAR through PAR binding motifs (PBMs) in the NLS. The elevation of the LLPS of TDP-43 following PAR binding in vitro was essential for TDP-43 aggregation in SGs in mammalian cells and neurons^132^. The findings indicate that when PARylation occurs at the TDP-43 NTD, highly negatively charged PAR biopolymers can induce LLPS, suggesting that LLPS occurs via multivalent interactions with the PBM in the N-terminal domain^132^.

## A novel PTM enhancing phase separation of ALS-associated RBPs

### Relationship between glutathionylation and phase separation in ALS-associated RBPs

Glutathione (GSH) is the most abundant thiol in all cells^133^. GSH can be converted to the oxidized form glutathione disulfide (GSSG), which is then converted back to GSH via nicotinamide adenine dinucleotide phosphate (NADPH)-dependent glutathione disulfide reductase (GSR)^134^. Glutathionylation is the most recently identified PTM that regulates the phase separation of ALS-associated RBPs. Protein glutathionylation is a major redox-sensitive PTM that can control the activity and stability of target proteins in response to cellular stress, including oxidative stress^135^. It is a reversible PTM on the cysteine thiol groups (-SH) of the substrate protein, formed via a disulfide bond with GSH^136^. Glutathionylation can occur via nonenzymatic or enzymatic reactions. Nonenzymatic glutathionylation occurs depending on the availability of GSH/GSSG; the process is nonspecific and typically proceeds under oxidative stress^137,138^. However, several enzymes, such as glutathione S-transferases (GSTs), have been proposed to catalyze glutathionylation^139^. GST pi (GSTP) is a class of GST that protects cells from reactive oxygen species by regulating GSH levels. GSTP has been implicated in glutathionylation^140,141^. However, glutathionylation is reversible through the release of GSH from cysteine residues in target proteins by thioredoxins and glutaredoxins, which are thiol oxidoreductases^138,142^. Interestingly, the GST omega (GSTO) class reportedly has the opposite effect from GSTP, inhibiting S-glutathionylation^143^.

**Fig. 2:**
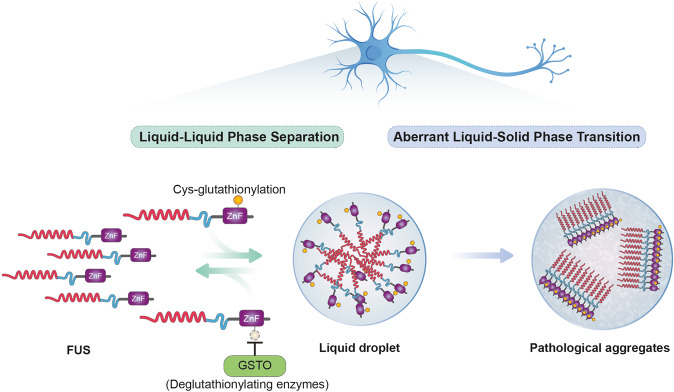
Glutathionylation of the cysteine residue of FUS in the zinc-finger (ZnF) domain promotes aberrant liquid‒liquid phase separation (LLPS). In the FUS-associated ALS disease model, glutathionylation of the FUS ZnF domain led to decreased FUS solubility by promoting phase separation and the formation of pathological aggregates. Glutathione S-transferase omega inhibits the phase separation of FUS via deglutathionylation to prevent the formation of pathological aggregates.

Several studies have suggested that the glutathionylation of specific proteins is significantly involved in the onset and progression of neurodegenerative diseases, including AD and ALS^144–146^. Another study that focused on identifying ALS biomarkers found that the deglutathionylating enzyme human GSTO1 was significantly reduced in peripheral blood mononuclear cells and spinal cord cells of sALS patients^147^. In our recent study, in addition to elucidating the pathogenic mechanism of FUS-associated ALS in both *Drosophila* and animal systems, we found that overexpression of GstO2, a *Drosophila* homolog of human GSTO1, reduces cytoplasmic FUS aggregates and attenuates neurodegenerative phenotypes, including mitochondrial dysfunction and neuronal toxicity^148^. The glutathionylation of the Cys447 residue on the ZnF domain of FUS when exposed to oxidative stress in vitro eventually leads to a decrease in FUS solubility by promoting phase separation and aggregate formation^148^. Interestingly, GstO2 inhibited FUS phase separation and aggregate formation by reducing glutathionylation in vitro and in *Drosophila* neurons (Fig. 2). Moreover, FUS-induced neuronal toxicity and cytoplasmic FUS accumulation are decreased by GSTO1 overexpression in mouse neuronal cells^148^. Accordingly, these findings suggest that the glutathionylation of FUS promotes phase separation and induces the formation of cytoplasmic aggregates. The suppression of glutathionylation is important in FUS-induced neurodegenerative diseases.

### Hypothesis concerning the molecular mechanism of glutathionylation-mediated phase separation

This section discusses the molecular mechanism by which cysteine glutathionylation regulates the phase separation of FUS and the physiological implications. Glutathionylation directly regulates the conformation and function of various proteins^149^. Many prior studies have addressed the functional changes of certain proteins via glutathionylation. However, little is known about glutathionylation-mediated conformational changes in proteins. Heat shock protein 70 (Hsp70) is a molecular chaperone that regulates protein homeostasis (proteostasis) by facilitating protein folding, handling misfolded proteins in cellular degradation pathways, and deterring protein aggregation^150^. Hsp70 has two cysteine residues (Cys574 and Cys603) in the C-terminal α-helical lid of the substrate-binding domain and undergoes glutathionylation^151^. Glutathionylation of these two residues leads to the unfolding of the α-helical lid structure. In contrast, deglutathionylation by dithiothreitol induces a reversible conformational change^151^. Similarly, another study revealed that binding immunoglobulin protein (BiP) also undergoes glutathionylation with an altered conformation. BiP is an Hsp70 chaperone located in the lumen of the endoplasmic reticulum. This chaperone is pivotal in protein folding and acts as the primary sensor in the activation of the unfolded protein response^152^. Glutathionylation of the Cys41 and Cys420 residues of BiP regulates the balance between foldase and ATPase activities by altering the protein structure. Interestingly, this modification led to decreased α-helix and increased β-sheet structure in BiP^153^. Stromal interaction molecule 1 (STIM1) is an ER-calcium (Ca^2+^)-sensing protein that regulates store-operated Ca^2+^ entry (SOCE) and other ion channels^154^. STIM1 was found to be glutathionylated at Cys49 or Cys56 residues, which are located near the EF-hand motif and sterile-α motif domain^155^. This modification induces thermodynamic destabilization and conformational changes, eventually resulting in increased solvent-exposed hydrophobicity^155^. Similarly, the glutathionylation of Cys56 leads to structural perturbations of the α-helix structure, such as the canonical EF-hand (i.e., α1 helix), α3, and α4 helices of the noncanonical EF-hand and the α6 and α8 helices of the sterile-α motif domain^155^. Taken together, these findings suggest that glutathionylation tends to induce a conformational change that unfolds the α-helical structure. FUS has a ZnF domain with two β-sheets and one α-helix. Moreover, FUS is glutathionylated at the Cys447 residue on the ZnF domain^148^. This suggests that glutathionylation of FUS may lead to a conformational change that unfolds the α-helix structure. This unfolding would allow FUS to maintain only its primary structure, providing an opportunity to interact with macromolecules, including RNA. Furthermore, glutathionylation adds a negative charge to the cysteine residue, which should affect multivalent interactions between molecules. It is assumed that this ultimately leads to the phase separation of FUS. However, the exact molecular mechanisms of the involvement glutathionylation in protein aggregate formation remain unclear. Further studies are needed to determine how the glutathionylation of RBP proteins occurs during the progression of ALS and how it is linked to the pathogenesis of ALS.

## Concluding remarks

**Fig. 3:**
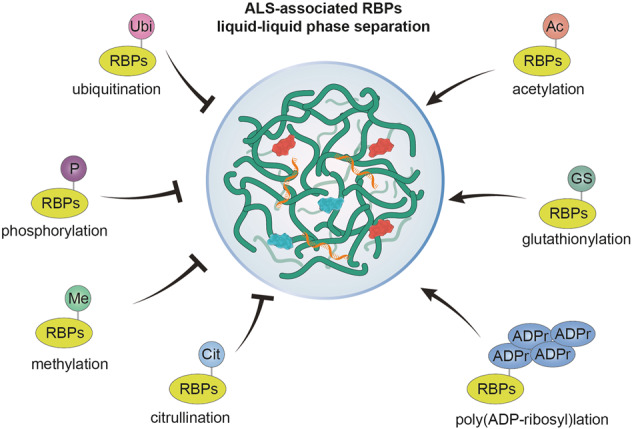
Effects of various posttranslational modifications (PTMs) on the phase separation of ALS-associated RNA-binding proteins (RBPs). PTMs can positively or negatively regulate LLPS in ALS-associated RBPs. Phosphorylation, methylation, citrullination, and ubiquitination of ALS-associated RBPs inhibit LLPS, whereas acetylation, poly(ADP-ribosyl)lation, and glutathionylation promote LLPS.

Many RBPs, such as FUS, TAF15, TDP-43, and hnRNPs, can undergo spontaneous and continuous LLPS and cytoplasmic aggregation in vitro and in vivo. Their assembly and transition to other phases must be tightly modulated in neurons. This review has discussed the molecular mechanisms of the regulation of protein aggregate formation by the PTMs of RBPs. The review highlights the important role of the recently described process of glutathionylation on protein aggregation in ALS pathogenesis. PTMs, including phosphorylation, methylation, citrullination, acetylation, ubiquitination, and PARylation, influence phase transition by modulating the structure, charge, hydrophobicity, and multivalent interactions that drive their phase separation (Fig. 3). This irreversible aggregation induced by phase separation changes RBPs to insoluble fibrils, which may be an important cause of RBP-linked proteinopathies. However, despite various studies of the mechanisms underlying LLPS regulation by PTMs, the specific regulators of the PTMs of RBPs remain unclear. Increased characterization and identification of novel PTMs that regulate the pathophysiological functions of RBPs will improve our ability to discern the pathological and physiological characteristics of RBPs in the development and progression of ALS.

In a recent study, in addition to elucidating the mechanism of phase transition regulation by a newly identified PTM, namely, the glutathionylation of FUS in both *Drosophila* and mammalian ALS models, we discovered that GSTO1/GstO2 regulates FUS status. FUS glutathionylation adds a negative charge to the cysteine residue of the FUS protein, which affects multivalent interactions between molecules. Weak intermolecular interactions, including electrostatic and hydrophobic interactions, could mediate the formation of FUS liquid droplets. Deglutathionylation of FUS, a protein causally associated with ALS, diminishes its LLPS and reduces abnormal cytoplasmic aggregates under prolonged stress, which may contribute to ALS pathogenesis. Given that cytoplasmic mislocalization and aggregate formation of FUS are common in neurons in FUS-associated proteinopathies, it is likely that regulation of the FUS glutathionylation-mediated stress-mitigating mechanism by GSTOs may also underlie other ALS cases and related diseases, including FTD. Although research on LLPS regulated by glutathionylation is still in its initial stages, the regulation of LLPS by this PTM will be a focus of future research on ALS. The detection of glutathionylated RBPs as disease markers may benefit many patients with ALS or related diseases. This PTM-related research will facilitate future clinical applications of phase transition for RBPs.

## References

[1] Rosen DR (1993). Mutations in Cu/Zn superoxide dismutase gene are associated with familial amyotrophic lateral sclerosis. Nature.

[2] Rowland LP, Shneider NA (2001). Amyotrophic lateral sclerosis. N. Engl. J. Med..

[3] Alonso A, Logroscino G, Jick SS, Hernán MA (2009). Incidence and lifetime risk of motor neuron disease in the United Kingdom: a population‐based study. Eur. J. Neurol..

[4] Cleveland DW, Rothstein JD (2001). From Charcot to Lou Gehrig: deciphering selective motor neuron death in ALS. Nat. Rev. Neurosci..

[5] Kapeli K, Martinez FJ, Yeo GW (2017). Genetic mutations in RNA-binding proteins and their roles in ALS. Hum. Genet..

[6] Zhao M, Kim JR, van Bruggen R, Park J (2018). RNA-binding proteins in amyotrophic lateral sclerosis. Mol. Cells.

[7] Brown RH, Al-Chalabi A (2017). Amyotrophic lateral sclerosis. N. Engl. J. Med..

[8] Forman MS, Trojanowski JQ, Lee VM (2004). Neurodegenerative diseases: a decade of discoveries paves the way for therapeutic breakthroughs. Nat. Med..

[9] Ross CA, Poirier MA (2004). Protein aggregation and neurodegenerative disease. Nat. Med..

[10] Chiti F, Dobson CM (2006). Protein misfolding, functional amyloid, and human disease. Annu. Rev. Biochem..

[11] Shin Y, Brangwynne CP (2017). Liquid phase condensation in cell physiology and disease. Science.

[12] Banani SF, Lee HO, Hyman AA, Rosen MK (2017). Biomolecular condensates: organizers of cellular biochemistry. Nat. Rev. Mol. Cell Biol..

[13] Zhang H (2020). Liquid-liquid phase separation in biology: mechanisms, physiological functions and human diseases. Sci. China Life Sci..

[14] Buchan JR, Parker R (2009). Eukaryotic stress granules: the ins and outs of translation. Mol. Cell.

[15] Fong K-w (2013). Whole-genome screening identifies proteins localized to distinct nuclear bodies. J. Cell Biol..

[16] Mitrea DM, Kriwacki RW (2016). Phase separation in biology; functional organization of a higher order. Cell Commun. Signal..

[17] Gomes E, Shorter J (2019). The molecular language of membraneless organelles. J. Biol. Chem..

[18] Sehgal PB, Westley J, Lerea KM, DiSenso-Browne S, Etlinger JD (2020). Biomolecular condensates in cell biology and virology: phase-separated membraneless organelles (MLOs). Anal. Biochem..

[19] Rai AK, Chen J-X, Selbach M, Pelkmans L (2018). Kinase-controlled phase transition of membraneless organelles in mitosis. Nature.

[20] Wang Z, Zhang H (2019). Phase separation, transition, and autophagic degradation of proteins in development and pathogenesis. Trends Cell Biol..

[21] Ramaswami M, Taylor JP, Parker R (2013). Altered ribostasis: RNA-protein granules in degenerative disorders. Cell.

[22] Antonicka H, Shoubridge EA (2015). Mitochondrial RNA granules are centers for posttranscriptional RNA processing and ribosome biogenesis. Cell Rep..

[23] Aguzzi A, Altmeyer M (2016). Phase separation: linking cellular compartmentalization to disease. Trends Cell Biol..

[24] Nedelsky NB, Taylor JP (2019). Bridging biophysics and neurology: aberrant phase transitions in neurodegenerative disease. Nat. Rev. Neurol..

[25] Ryan VH, Fawzi NL (2019). Physiological, pathological, and targetable membraneless organelles in neurons. Trends Neurosci..

[26] Zbinden A, Perez-Berlanga M, De Rossi P, Polymenidou M (2020). Phase separation and neurodegenerative diseases: a disturbance in the force. Dev. Cell.

[27] Alberti S, Gladfelter A, Mittag T (2019). Considerations and challenges in studying liquid-liquid phase separation and biomolecular condensates. Cell.

[28] Duan G, Walther D (2015). The roles of post-translational modifications in the context of protein interaction networks. PLoS Comput. Biol..

[29] Prabakaran S, Lippens G, Steen H, Gunawardena J (2012). Post-translational modification: nature’s escape from genetic imprisonment and the basis for dynamic information encoding. Wires Syst. Biol. Med..

[30] Snead WT, Gladfelter AS (2019). The control centers of biomolecular phase separation: how membrane surfaces, PTMs, and active processes regulate condensation. Mol. Cell.

[31] Luo Y-Y, Wu J-J, Li Y-M (2021). Regulation of liquid-liquid phase separation with focus on post-translational modifications. Chem. Commun..

[32] Owen I, Shewmaker F (2019). The role of post-translational modifications in the phase transitions of intrinsically disordered proteins. Int. J. Mol. Sci..

[33] Ong JY, Torres JZ (2020). Phase separation in cell division. Mol. Cell.

[34] Molliex A (2015). Phase separation by low complexity domains promotes stress granule assembly and drives pathological fibrillization. Cell.

[35] Markmiller S (2018). Context-dependent and disease-specific diversity in protein interactions within stress granules. Cell.

[36] Babinchak WM (2019). The role of liquid–liquid phase separation in aggregation of the TDP-43 low-complexity domain. J. Biol. Chem..

[37] Murthy AC (2019). Molecular interactions underlying liquid−liquid phase separation of the FUS low-complexity domain. Nat. Struct. Mol. Biol..

[38] Alberti S, Halfmann R, King O, Kapila A, Lindquist S (2009). A systematic survey identifies prions and illuminates sequence features of prionogenic proteins. Cell.

[39] Kato M (2012). Cell-free formation of RNA granules: low complexity sequence domains form dynamic fibers within hydrogels. Cell.

[40] Malinovska L, Palm S, Gibson K, Verbavatz J-M, Alberti S (2015). Dictyostelium discoideum has a highly Q/N-rich proteome and shows an unusual resilience to protein aggregation. Proc. Natl Acad. Sci. USA.

[41] Dyson HJ (2011). Expanding the proteome: disordered and alternatively folded proteins. Q. Rev. Biophys..

[42] Li P (2012). Phase transitions in the assembly of multivalent signalling proteins. Nature.

[43] Wang J (2018). A molecular grammar governing the driving forces for phase separation of prion-like RNA binding proteins. Cell.

[44] Nott TJ (2015). Phase transition of a disordered nuage protein generates environmentally responsive membraneless organelles. Mol. Cell.

[45] Kim S (2016). Complexation and coacervation of like-charged polyelectrolytes inspired by mussels. Proc. Natl Acad. Sci. USA.

[46] Pak CW (2016). Sequence determinants of intracellular phase separation by complex coacervation of a disordered protein. Mol. Cell.

[47] Kang J, Lim L, Lu Y, Song J (2019). A unified mechanism for LLPS of ALS/FTLD-causing FUS as well as its modulation by ATP and oligonucleic acids. PLoS Biol..

[48] Das S, Lin Y-H, Vernon RM, Forman-Kay JD, Chan HS (2020). Comparative roles of charge, π, and hydrophobic interactions in sequence-dependent phase separation of intrinsically disordered proteins. Proc. Natl Acad. Sci. USA.

[49] Brangwynne CP, Tompa P, Pappu RV (2015). Polymer physics of intracellular phase transitions. Nat. Phys..

[50] Boeynaems S (2018). Protein phase separation: a new phase in cell biology. Trends Cell Biol..

[51] Johnson BS (2009). TDP-43 is intrinsically aggregation-prone, and amyotrophic lateral sclerosis-linked mutations accelerate aggregation and increase toxicity. J. Biol. Chem..

[52] Conicella AE, Zerze GH, Mittal J, Fawzi NL (2016). ALS mutations disrupt phase separation mediated by α-helical structure in the TDP-43 low-complexity C-terminal domain. Structure.

[53] Schmidt HB, Rohatgi R (2016). In vivo formation of vacuolated multi-phase compartments lacking membranes. Cell Rep..

[54] Mackenzie IR (2017). TIA1 mutations in amyotrophic lateral sclerosis and frontotemporal dementia promote phase separation and alter stress granule dynamics. Neuron.

[55] Dao TP (2019). ALS-linked mutations affect UBQLN2 oligomerization and phase separation in a position-and amino acid-dependent manner. Structure.

[56] Ding X, Gu S, Xue S, Luo S-Z (2021). Disease-associated mutations affect TIA1 phase separation and aggregation in a proline-dependent manner. Brain Res..

[57] Grese ZR (2021). Specific RNA interactions promote TDP‐43 multivalent phase separation and maintain liquid properties. EMBO Rep..

[58] Andersson MK (2008). The multifunctional FUS, EWS and TAF15 proto-oncoproteins show cell type-specific expression patterns and involvement in cell spreading and stress response. BMC Cell Biol..

[59] Colombrita C (2009). TDP‐43 is recruited to stress granules in conditions of oxidative insult. J. Neurochem.

[60] Tan AY, Manley JL (2009). The TET family of proteins: functions and roles in disease. J. Mol. Cell Biol..

[61] March ZM, King OD, Shorter J (2016). Prion-like domains as epigenetic regulators, scaffolds for subcellular organization, and drivers of neurodegenerative disease. Brain Res..

[62] Loughlin FE (2019). The solution structure of FUS bound to RNA reveals a bipartite mode of RNA recognition with both sequence and shape specificity. Mol. Cell.

[63] Luo F (2018). Atomic structures of FUS LC domain segments reveal bases for reversible amyloid fibril formation. Nat. Struct. Mol. Biol..

[64] Murray DT (2017). Structure of FUS protein fibrils and its relevance to self-assembly and phase separation of low-complexity domains. Cell.

[65] Murray DT, Tycko R (2020). Side chain hydrogen-bonding interactions within amyloid-like fibrils formed by the low-complexity domain of FUS: evidence from solid state nuclear magnetic resonance spectroscopy. Biochemistry.

[66] Lee M, Ghosh U, Thurber KR, Kato M, Tycko R (2020). Molecular structure and interactions within amyloid-like fibrils formed by a low-complexity protein sequence from FUS. Nat. Commun..

[67] Patel A (2015). A liquid-to-solid phase transition of the ALS protein FUS accelerated by disease mutation. Cell.

[68] Ding X (2020). Amyloid-forming segment induces aggregation of FUS-LC domain from phase separation modulated by site-specific phosphorylation. J. Mol. Biol..

[69] Chen J (2022). The SGYS motif of TAF15 prion-like domain is critical to amyloid fibril formation. Biophys. J..

[70] Nosella ML (2021). O-linked-N-acetylglucosaminylation of the RNA-binding protein EWS N-terminal low complexity region reduces phase separation and enhances condensate dynamics. J. Am. Chem. Soc..

[71] Chong S (2022). Tuning levels of low-complexity domain interactions to modulate endogenous oncogenic transcription. Mol. Cell.

[72] Mompeán M (2016). The TDP‐43 N‐terminal domain structure at high resolution. FEBS J..

[73] Chang C-k (2012). The N-terminus of TDP-43 promotes its oligomerization and enhances DNA binding affinity. Biochem. Biophys. Res. Commun..

[74] D’Ambrogio A (2009). Functional mapping of the interaction between TDP-43 and hnRNP A2 in vivo. Nucleic Acids Res.

[75] Romano M (2014). Evolutionarily conserved heterogeneous nuclear ribonucleoprotein (hnRNP) A/B proteins functionally interact with human and Drosophila TAR DNA-binding protein 43 (TDP-43). J. Biol. Chem..

[76] Geuens T, Bouhy D, Timmerman V (2016). The hnRNP family: insights into their role in health and disease. Hum. Genet..

[77] Kim HJ (2013). Mutations in prion-like domains in hnRNPA2B1 and hnRNPA1 cause multisystem proteinopathy and ALS. Nature.

[78] Han TW (2012). Cell-free formation of RNA granules: bound RNAs identify features and components of cellular assemblies. Cell.

[79] Ma M-R, Hu Z-W, Zhao Y-F, Chen Y-X, Li Y-M (2016). Phosphorylation induces distinct alpha-synuclein strain formation. Sci. Rep..

[80] Hu Z-W (2017). Phosphorylation at Ser8 as an intrinsic regulatory switch to regulate the morphologies and structures of Alzheimer’s 40-residue β-amyloid (Aβ40) fibrils. J. Biol. Chem..

[81] Hofweber M, Dormann D (2019). Friend or foe—post-translational modifications as regulators of phase separation and RNP granule dynamics. J. Biol. Chem..

[82] Monahan Z (2017). Phosphorylation of the FUS low‐complexity domain disrupts phase separation, aggregation, and toxicity. EMBO J..

[83] Lin Y, Currie SL, Rosen MK (2017). Intrinsically disordered sequences enable modulation of protein phase separation through distributed tyrosine motifs. J. Biol. Chem..

[84] Wang A (2018). A single N‐terminal phosphomimic disrupts TDP‐43 polymerization, phase separation, and RNA splicing. EMBO J..

[85] Hornbeck PV (2012). PhosphoSitePlus: a comprehensive resource for investigating the structure and function of experimentally determined post-translational modifications in man and mouse. Nucleic Acids Res..

[86] Hornbeck PV (2015). PhosphoSitePlus, 2014: mutations, PTMs and recalibrations. Nucleic Acids Res.

[87] Neumann M (2009). Phosphorylation of S409/410 of TDP-43 is a consistent feature in all sporadic and familial forms of TDP-43 proteinopathies. Acta Neuropathol..

[88] Brady OA, Meng P, Zheng Y, Mao Y, Hu F (2011). Regulation of TDP‐43 aggregation by phosphorylation andp62/SQSTM1. J. Neurochem..

[89] Kametani F (2009). Identification of casein kinase-1 phosphorylation sites on TDP-43. Biochem. Biophys. Res. Commun..

[90] Gruijs da Silva LA (2022). Disease‐linked TDP‐43 hyperphosphorylation suppresses TDP‐43 condensation and aggregation. EMBO J..

[91] Kametani F (2016). Mass spectrometric analysis of accumulated TDP-43 in amyotrophic lateral sclerosis brains. Sci. Rep..

[92] Ryan VH (2021). Tyrosine phosphorylation regulates hnRNPA2 granule protein partitioning and reduces neurodegeneration. EMBO J..

[93] Lao Z (2022). Insights into the atomistic mechanisms of phosphorylation in disrupting liquid–liquid phase separation and aggregation of the FUS low-complexity domain. J. Chem. Inf. Model..

[94] Vernon RM (2018). Pi-Pi contacts are an overlooked protein feature relevant to phase separation. eLife.

[95] Bedford MT, Richard S (2005). Arginine methylation: an emerging regulatorof protein function. Mol. Cell.

[96] Bedford MT, Clarke SG (2009). Protein arginine methylation in mammals: who, what, and why. Mol. Cell.

[97] Gayatri S, Bedford MT (2014). Readers of histone methylarginine marks. BBA Gene Regul. Mech..

[98] Lorton BM, Shechter D (2019). Cellular consequences of arginine methylation. Cell. Mol. Life Sci..

[99] Guccione E, Richard S (2019). The regulation, functions and clinical relevance of arginine methylation. Nat. Rev. Mol. Cell Biol..

[100] Rappsilber J, Friesen WJ, Paushkin S, Dreyfuss G, Mann M (2003). Detection of arginine dimethylated peptides by parallel precursor ion scanning mass spectrometry in positive ion mode. Anal. Chem..

[101] Dormann D (2012). Arginine methylation next to the PY‐NLS modulates Transportin binding and nuclear import of FUS. EMBO J..

[102] Neumann M (2012). Transportin 1 accumulates specifically with FET proteins but no other transportin cargos in FTLD-FUS and is absent in FUS inclusions in ALS with FUS mutations. Acta Neuropathol..

[103] Qamar S (2018). FUS phase separation is modulated by a molecular chaperone and methylation of arginine cation-π interactions. Cell.

[104] Hofweber M (2018). Phase separation of FUS is suppressed by its nuclear import receptor and arginine methylation. Cell.

[105] Friend LR (2013). Arginine methylation of hnRNP A2 does not directly govern its subcellular localization. PloS One.

[106] Ryan VH (2018). Mechanistic view of hnRNPA2 low-complexity domain structure, interactions, and phase separation altered by mutation and arginine methylation. Mol. Cell.

[107] Rogers G, Simmonds D (1958). Content of citrulline and other amino-acids in a protein of hair follicles. Nature.

[108] Rogers G (1962). Occurrence of citrulline in proteins. Nature.

[109] Anzilotti C, Pratesi F, Tommasi C, Migliorini P (2010). Peptidylarginine deiminase 4 and citrullination in health and disease. Autoimmun. Rev..

[110] Mondal S, Thompson PR (2019). Protein arginine deiminases (PADs): biochemistry and chemical biology of protein citrullination. Acc. Chem. Res..

[111] Knuckley B (2010). Substrate specificity and kinetic studies of PADs 1, 3, and 4 identify potent and selective inhibitors of protein arginine deiminase 3. Biochemistry.

[112] Tarcsa E (1996). Protein unfolding by peptidylarginine deiminase: substrate specificity and structural relationships of the natural substrates trichohyalin and filaggrin. J. Biol. Chem..

[113] Tanikawa C (2018). Citrullination of RGG motifs in FET proteins by PAD4 regulates protein aggregation and ALS susceptibility. Cell Rep..

[114] Drazic A, Myklebust LM, Ree R, Arnesen T (2016). The world of protein acetylation. BBA-Proteins Proteom..

[115] Narita T, Weinert BT, Choudhary C (2019). Functions and mechanisms of non-histone protein acetylation. Nat. Rev. Mol. Cell Biol..

[116] Zhao S (2010). Regulation of cellular metabolism by protein lysine acetylation. Science.

[117] Yang X-J, Seto E (2008). Lysine acetylation: codified crosstalk with other posttranslational modifications. Mol. Cell.

[118] Xia C, Tao Y, Li M, Che T, Qu J (2020). Protein acetylation and deacetylation: An important regulatory modification in gene transcription. Exp. Ther. Med..

[119] Bock AS (2021). N‐terminal acetylation modestly enhances phase separation and reduces aggregation of the low‐complexity domain of RNA‐binding protein fused in sarcoma. Protein Sci..

[120] Hershko A, Ciechanover A (1998). The ubiquitin system. Annu. Rev. Biochem..

[121] Nandi D, Tahiliani P, Kumar A, Chandu D (2006). The ubiquitin-proteasome system. J. Biosci..

[122] Zheng Q (2016). Dysregulation of ubiquitin-proteasome system in neurodegenerative diseases. Front. Aging Neurosci..

[123] Mani A, Gelmann EP (2005). The ubiquitin-proteasome pathway and its role in cancer. J. Clin. Oncol..

[124] Kwon S, Zhang Y, Matthias P (2007). The deacetylase HDAC6 is a novel critical component of stress granules involved in the stress response. Genes Dev..

[125] Wu A-L, Wang J, Zheleznyak A, Brown EJ (1999). Ubiquitin-related proteins regulate interaction of vimentin intermediate filaments with the plasma membrane. Mol. Cell.

[126] Deng H-X (2011). Mutations in UBQLN2 cause dominant X-linked juvenile and adult-onset ALS and ALS/dementia. Nature.

[127] Dao TP (2018). Ubiquitin modulates liquid-liquid phase separation of UBQLN2 via disruption of multivalent interactions. Mol. Cell.

[128] Alemasova EE, Lavrik OI (2019). Poly (ADP-ribosyl) ation by PARP1: reaction mechanism and regulatory proteins. Nucleic Acids Res..

[129] Leung AK (2011). Poly (ADP-ribose) regulates stress responses and microRNA activity in the cytoplasm. Mol. Cell.

[130] Altmeyer M (2015). Liquid demixing of intrinsically disordered proteins is seeded by poly (ADP-ribose). Nat. Commun..

[131] Duan Y (2019). PARylation regulates stress granule dynamics, phase separation, and neurotoxicity of disease-related RNA-binding proteins. Cell Res..

[132] McGurk L (2018). Poly (ADP-ribose) prevents pathological phase separation of TDP-43 by promoting liquid demixing and stress granule localization. Mol. Cell.

[133] Meister A (1988). Glutathione metabolism and its selective modification. J. Biol. Chem..

[134] Anderson ME (1998). Glutathione: an overview of biosynthesis and modulation. Chem. Biol. Interact..

[135] Mieyal JJ, Gallogly MM, Qanungo S, Sabens EA, Shelton MD (2008). Molecular mechanisms and clinical implications of reversible protein S-glutathionylation. Antioxid. Redox Signal..

[136] Dalle-Donne I, Rossi R, Giustarini D, Colombo R, Milzani A (2007). S-glutathionylation in protein redox regulation. Free Radic. Biol. Med..

[137] Ziegler D (1985). Role of reversible oxidation-reduction of enzyme thiols-disulfides in metabolic regulation. Annu. Rev. Biochem..

[138] Gallogly MM, Mieyal JJ (2007). Mechanisms of reversible protein glutathionylation in redox signaling and oxidative stress. Curr. Opin. Pharmacol..

[139] Klaus A (2013). Glutathione S-transferases interact with AMP-activated protein kinase: evidence for S-glutathionylation and activation in vitro. PLoS ONE.

[140] Baez S, Segura-Aguilar J, Widersten M, Johansson A-S, Mannervik B (1997). Glutathione transferases catalyse the detoxication of oxidized metabolites (o-quinones) of catecholamines and may serve as an antioxidant system preventing degenerative cellular processes. Biochem. J..

[141] Tew KD, Ronai ZE (1999). GST function in drug and stress response. Drug Resist. Update.

[142] Ghezzi P, Bonetto V, Fratelli M (2005). Thiol–disulfide balance: from the concept of oxidative stress to that of redox regulation. Antioxid. Redox Signal..

[143] Menon D, Board PG (2013). A role for glutathione transferase Omega 1 (GSTO1-1) in the glutathionylation cycle. J. Biol. Chem..

[144] Sabens Liedhegner EA, Gao X-H, Mieyal JJ (2012). Mechanisms of altered redox regulation in neurodegenerative diseases—focus on S-glutathionylation. Antioxid. Redox Signal..

[145] Halloran M, Parakh S, Atkin J (2013). The role of s-nitrosylation and s-glutathionylation of protein disulphide isomerase in protein misfolding and neurodegeneration. Int. J. Cell Biol..

[146] Gorelenkova Miller O, Mieyal JJ (2015). Sulfhydryl-mediated redox signaling in inflammation: role in neurodegenerative diseases. Arch. Toxicol..

[147] Nardo G (2011). Amyotrophic lateral sclerosis multiprotein biomarkers in peripheral blood mononuclear cells. PLoS ONE.

[148] Cha SJ (2022). Therapeutic modulation of GSTO activity rescues FUS-associated neurotoxicity via deglutathionylation in ALS disease models. Dev. Cell.

[149] Zhang J, Ye Z-w, Singh S, Townsend DM, Tew KD (2018). An evolving understanding of the S-glutathionylation cycle in pathways of redox regulation. Free Radic. Biol. Med..

[150] Porter CM, Truman AW, Truttmann MC (2020). Post-translational modifications of Hsp70 family proteins: Expanding the chaperone code. J. Biol. Chem..

[151] Yang J (2020). S-Glutathionylation of human inducible Hsp70 reveals a regulatory mechanism involving the C-terminal α-helical lid. J. Biol. Chem..

[152] Gething M-J (1999). Role and regulation of the ER chaperone BiP. Semin. Cell Dev. Biol..

[153] Zhang J (2020). Altered redox regulation and S-glutathionylation of BiP contribute to bortezomib resistance in multiple myeloma. Free Radic. Biol. Med..

[154] Liou J (2005). STIM is a Ca2+ sensor essential for Ca2+-store-depletion-triggered Ca2+ influx. Curr. Biol..

[155] Sirko C, Novello MJ, Stathopulos PB (2022). An S-glutathiomimetic provides structural insights into stromal interaction molecule-1 regulation. J. Mol. Biol..

